# Antimicrobial activity of nano cerium oxide (IV) (CeO2) against *Streptococcus mutans*

**DOI:** 10.1186/1753-6561-8-S4-P48

**Published:** 2014-10-01

**Authors:** Carlos Christiano Lima dos Santos, Isabela Albuquerque Passos Farias, Allan de Jesus dos Reis Albuquerque, Patrícia Maria de Freitas e Silva, Giselle Medeiros da Costa One, Fabio Correia Sampaio

**Affiliations:** 1 Federal University of Paraiba, João Pessoa, Brasil; 2 State University of Paraiba, João Pessoa, Brasil

## Background

Cerium oxide (CeO_2_) is a technologically important material due to its properties and applications in several areas that range from engineering to biological sciences. Despite the hydrothermal microwave method versatility, its crystallization kinetics has low speed when the processing temperatures are below 300°C which are the desired conditions for soft chemistry processes [1,2]. At lower temperatures it was found that this material has antimicrobial activity against several bacteria, including *E. coli, B. subtilis, Shewanellaoneidensis *and *Pseudokirchneriella subcapitata*, destroying microrganisms cell walls by probable action of reactive oxygen species [3]. When nanoparticles of CeO_2 _crystals are synthesized (IV), the antimicrobial activity can be better controlled and its spectrum of applications expanded.

## Methods

In this work, cerium oxide nanoparticles (IV) were synthesized by the hydrothermal method, assisted by microwave, with a 150°C temperature, approximately, at intervals of 10, 15 and 20 minutes. Their structural, morphological and microbiological characteristics were studied by x-ray diffraction (XRD), high-resolution electronic scanning microscopy, (FEG-SEM), electronic microscopy of transmission (TEM), BET method, infrared spectroscopy (IR), resazurin, spectrophotometry, fluorescence and agar diffusion.

## Results

All XRD samples showed peaks corresponding to planes (1 1 1), (2 0 0), (0 2 2), (3 1 1), (2 2 2) and (4 0 0) of a centered face cube, fluorite, attributed to CeO2 (I) according to law JCPDS 34-0394. Broad reflection peaks were clearly visible, demonstrating that the crystal size is small and must be in the nanoscale dimension, which indicates the efficiency of the method for obtaining this nanostructured material. Infrared spectra showed a band of vibration between 700 and 500 cm -1 related to the vibration of the metal- oxygen (Ce-O) and other results. Their surface areas were approximately 200m^2 ^/ g with around particle sizes of 5nm. Micrographs showed different sizes and shapes of clusters. Microbiological tests against *Streptococcus mutans *for the containing nano cerium oxide (IV) composite in ZnO at 10% (diluted in BHI- broth) showed antibacterial effect in minimum inhibitory concentration of 0.22 mg / mL.

## Conclusions

The results showed the direct influence of the synthesis method employed on the morphology and size of nanoparticles cerium oxide (IV) obtained. Nano cerium oxide (IV) seems to be a material of great potential for use against *S. mutans *(a dental bacteria) responsible for cariogenic process due to its purity and particle size. The matching results among the tests employed in biological assays showed that the techniques of fluorescence, spectrophotometry, resazurin and fluorescence did not suffer reagent interference as may eventually occur with some products of natural origin. The dental composite consisting of odontologic ZnO and nanometer CeO_2 _in the proportion of 10 % showed superior antimicrobial properties when compared to the standard used (dental zinc oxide + eugenol).
